# Debating the Future of Work: The Perception and Reaction of the Spanish Workforce to Digitization and Automation Technologies

**DOI:** 10.3389/fpsyg.2020.01965

**Published:** 2020-08-10

**Authors:** Carolina Rodriguez-Bustelo, Joan Manuel Batista-Foguet, Ricard Serlavós

**Affiliations:** Esade, Universitat Ramon Llull, Barcelona, Spain

**Keywords:** structural equation models, Spanish workforce, preparation for the work automatization, fear to the digital work environment, education for the future, complexity of the work

## Abstract

Given the significant changes that are expected in the nature of work as a consequence of rapid technological advances, it is crucial that society finds ways to maximize benefits while recognizing and mitigating related challenges. This article is intended to fill a current research gap in this context by examining how aware and prepared affected workers are for the challenges predicted by research. This information is crucial since expectation and preparation of the workforce will significantly influence society’s adaptability to the future. As a result of the article various significant relationships among workers’ characteristics and their attitude towards automation could be identified. The interviewed workers’ level of fear appears to have very little influence on preparation for automation-driven changes in the future while perceived opportunity significantly impacts this degree of preparation. Characteristics that additionally most influence the degree of preparatory steps taken by respondents are their level of education as well as work complexity and position. These findings should be used to identify potential ways for relevant stakeholders to adequately prepare for and meet the challenges of the impending increase of automation in the workplace.

## Introduction

The rise of new technologies such as artificial intelligence, smart technology, automation and robotics is predicted to practically affect all aspects of our society, lives and economy substantially (Makridakis, 2017). While some of the changes will undoubtedly improve our lives, the nature of the expected impact of the digital revolution on labor and employment is controversial. Research has shown that a reduction in demand for labor and in wages overall as well as significant qualitative changes in the labor market can be expected (Freddi, 2017; Acemoglu and Restrepo, 2018) which might lead to a mismatch between available and required skills for the workforce of the future.

Many automatable jobs will become obsolete, and this will affect not only manufacturing jobs but also what are today commonly viewed as “white-collar” jobs such as customer service or administrative tasks (Smith and Anderson, 2014). Some studies have found that about half of all current jobs in advanced economies are at risk of being automated within the next 10–20 years (Dengler and Matthes, 2018). While this number is frequently challenged and opinions in research vary (World Economic Forum, 2016) it is clear that today’s workforce and the quality of work will undergo a significant change in the near future (Spencer, 2018).

This prediction poses the threat of high unemployment as well as an increase in wealth inequality in the affected societies (Makridakis, 2017). However, AI technologies provide vast opportunities for new products and services as well as immense productivity improvements and are indispensable in modern economies (ITU, 2018). One of the greatest challenge societies and firms therefore face today is how to best utilize those benefits while avoiding the risks and disadvantages. Alongside the rapid and often uncertain advances in technology, there is the long-term, predictable trend of aging populations and workforces across the globe. The simultaneous trends of increased longevity along with rapid technological innovation will significantly change the way we work in the future, posing challenges for governments, businesses and individuals alike (Lewis, 2020). Society will most likely need to adopt policies and reforms to protect companies, individuals and families from negative consequences of changes in the future of work.

It is in this context that it is essential to gain insight into how aware the affected workers are of the challenges predicted by research because this awareness could significantly influence not only the preparation and actions the workforce might be willing to take, but also the effectiveness and adequateness of policies and other measures to be implemented. Even though one would expect the view of the workers to be essential in identifying appropriate solutions, little attention seems to have been directed toward their expectations or the potential impact thereof in most of the recent research on the issue of AI, automation and the future of work.

The objective of this article therefore is to determine – on the basis of a survey conducted by the Future for Work Institute in Barcelona – how concerned the Spanish workforce is regarding the consequences of automation and regarding potential job loss and which steps it is looking to take in preparation. This article further aims to determine whether the actual perception of automation by a sample of the Spanish workforce is adequately reflected in or rather diverges from current literature and research on the issue. Learning how aware the workforce is of the challenges laying ahead is highly relevant in order to identify the correct measures both from a legislative as well as an individual employer and industry perspective.

If the needed awareness and willingness to adapt to future requirements is missing or misdirected in a workforce, well intended policies or re-training measures that rely on this awareness might prove useless or even backfire. Closing this research gap could therefore not only influence how effectively workers are preparing for the future, but also how successfully policy makers and other stakeholders can assist them in doing so. The findings set out herein will therefore hopefully add value to the current discussion by guiding company and government efforts toward effective mitigative measures such as reskilling, education and policies by allowing them to take into account the indispensable perspective of the workforce and its characteristics.

## Estimated Impact of Technology on the Workforce

The current discussion on the future of work is largely dominated by considerations of the expected impact of new technologies such as driverless cars, smart factories or service robots that are powered by advances in robotics and artificial intelligence and have the ability to automate and replace human capabilities. These technologies are increasingly also being applied to domains and tasks that until very recently were believed to particularly require human capabilities such as reasoning, sensing, and deciding (Arntz et al., 2016). It is becoming increasingly clear that not only repetitive and low skills are at risk but that automation, robotics and AI can also provide equal or better services than humans as dermatologists, financial reporters or lawyers (Rainie and Anderson, 2017).

In 1984, Nils J Nilsson in “*Artificial Intelligence, Employment and Income*” already described the profound effects artificial intelligence would have on the nature of labor. In his analysis, he predicted that AI would drastically reduce the need for human labor and largely impact the distribution of income. He also discussed the fear people have of their work being replaced by machines and the following rise in unemployment. Nilsson, however, believed the identified apprehension to be somewhat paradoxical and emphasized the potential upsides of a reduction in needed labor: the population should rather be excited to have more free time for activities (Nilsson, 1984). More than 30 years later, first effects of automation and digitization can be observed, and general apprehension persists, but a more complex understanding has started to prevail, suggesting that automation will bring neither apocalypse nor utopia, but instead both benefits and stress to society (Muro et al., 2019).

Technology has already started to change the workforce with jobs vanishing at an accelerating rate (Kile, 2013). Smith and Anderson (2014) found the experts’ predictions to be largely consistent in that robotics and artificial intelligence would transform wide segments of daily life by 2025, with strong implications for a range of industries such as health care, transport and logistics or customer service. However, the same experts were divided on how these technological advances would influence the overall economic and employment landscape over the next decade (Smith and Anderson, 2014).

There is a general consensus among researchers that increasing replacement of human labor with machines reduces the demand for labor and wages (Brynjolfsson and McAfee, 2014; Acemoglu and Restrepo, 2018). The specific assessments regarding the extent of the impact on tasks, jobs or occupations being automated, however, vary widely. In a study determining affected occupations and analyzing the technological capability to replace them. Frey et al. estimated about 47 percent of total United States employment to be at risk (Frey et al., 2013) a prognosis which caused great upheaval and attention within academia as well as in the media. In a similar study in Europe, Bowles concluded that the percentage of jobs in Europe at risk of being replaced ranges between 45 to more than 60 percent (Bowles, 2014).

Other researchers, however, disagree with these empirical prognoses and believe them to be based on an overestimation of the potential of automation. One argument for this view is that the overly pessimistic scenarios disregard the heterogeneity of tasks within occupations as well as the adaptability of jobs in the digital transformation (Arntz et al., 2016). Individual tasks rather than entire occupations should accordingly be analyzed for their risk of being replaced. Arntz et al. (2016) examined the automation potential on this task level (instead of on an occupation level) and found that across the 21 OECD countries, only 9 percent of jobs should in fact be technically automatable and therefore at risk of becoming redundant. Supplementary Appendix S1 shows the percentage of workers with high automatability by OECD country, determining Austria, Germany and Spain to show the highest risk (Arntz et al., 2016).

From an economic point of view, however, machines of course cannot only replace, but also complement human labor. The cost-savings achieved through automation could also lead to an increase in productivity, growth and a higher demand for (non-automatable) labor (Autor, 2015). This would then presumably lead to an increase in both workers’ incomes and product demand. The expectation would appear to be supported by a McKinsey & Company study which found that only about six percent of companies expect their workforce in Europe and the United States to shrink as a result of automation and artificial intelligence, while more than 17 percent even expect their workforces to grow (Bughin et al., 2018).

Researchers who reach more positive outlooks further claim that the technical potential for automation is frequently confounded with employment loss and argue that technological possibility does not equal economic reality (Muro et al., 2019). The potential to use a machine for a certain task does not necessarily mean that the human worker will ultimately be replaced since there are often financial, ethical and legal obstacles involved (Arntz et al., 2016). Moreover, studies on automation usually only consider existing jobs, while the application of new technologies can also be expected to create new employment opportunities (Arntz et al., 2016). Some researchers accordingly believe that due to the inhibiting and balancing mechanisms mentioned above, technological advances will have a much stronger effect on the nature and composition of work than on the level of employment (Kapeliushnikov, 2019).

Acemoglu and Restrepo (2018) also stress that the countervailing effects are not fully balanced and synchronized. One of the biggest societal challenges will be adequate handling of the mismatch between the skills required in the context of new technologies and those being replaced (Acemoglu and Restrepo, 2018) meaning that the worker being replaced by an automated car assembly line might not easily be retrained to technically oversee that assembly line. Supplementary Appendix S2 gives an overview of the skill mismatches McKinsey & Company identified in their study which are expected to be most extreme when automation and AI technologies are adopted in certain work functions (Bughin et al., 2018).

The above discussion of various studies shows that current research on the subject of automation and the future of work should be interpreted with some caution. The existing studies reflect today’s technological capabilities based on experts’ assessments, rather than the actual application of the technologies in companies – which might lead to a general overestimation of job automatability. While it is certain that society will be impacted and that labor requirements will change, the decision to automate tasks and replace human labor will most certainly not only depend on technological capabilities. The actual effect on the nature of work will be determined significantly by the velocity in which technology is introduced to work environments as well as possible synergies of humans working with machines. In addition to economic factors, cultural, political, ethical and legal aspects need to be considered, for instance when letting algorithms make crucial decisions or assessing liabilities in the context of automation. Overall, the great challenge facing the future of work will foreseeably be a change in required skills and knowledge for large parts of the workforce rather than a significant change in overall employment levels.

## Estimated Impact by Level of Education and Type of Work

All of the reviewed researchers agree that the likelihood of a work task being automated depends on how easy it is to express the task in terms of coded rules and algorithms, since only those can be carried out easily by machines. Routine tasks (a certain process is repeated frequently with predictable deviations and outcomes) are easier to codify and therefore more likely to be technically substituted (Autor, 2015). Since carrying out routine codifiable tasks also tends to be related to less educational formation and lower income, the risk of work being automated increases for those workers with lower levels of education and income (Arntz et al., 2016).

According to companies’ own assessments, individuals with a college degree are more likely to be hired, receive retraining, and less likely to be dismissed than those without a degree (Bughin et al., 2018). A study by the Brookings Institution further determined that male, young and less educated workers, along with minority groups, are more likely to face challenges from automation in the next years (Muro et al., 2019). In their study (Van der Heijde et al., 2018), further show how important a company’s learning climate is for workers’ employability irrespective of life or career stage.

Frey et al. (2013) analyzed various occupations and assigned an employment risk category to each (see Supplementary Appendix S3). They concluded that the occupations most at risk are occupations in the service, sales, office and administrative, production as well as transportation and material moving industries. Occupations in management, business, financial, computer, engineering, science, education, legal, arts, media, healthcare and technical fields are less likely to be replaced by machines (Frey et al., 2013). As shown in Supplementary Appendix S4, the 2019 report by the Brookings Institution supports these findings (Muro et al., 2019).

**FIGURE 1:**
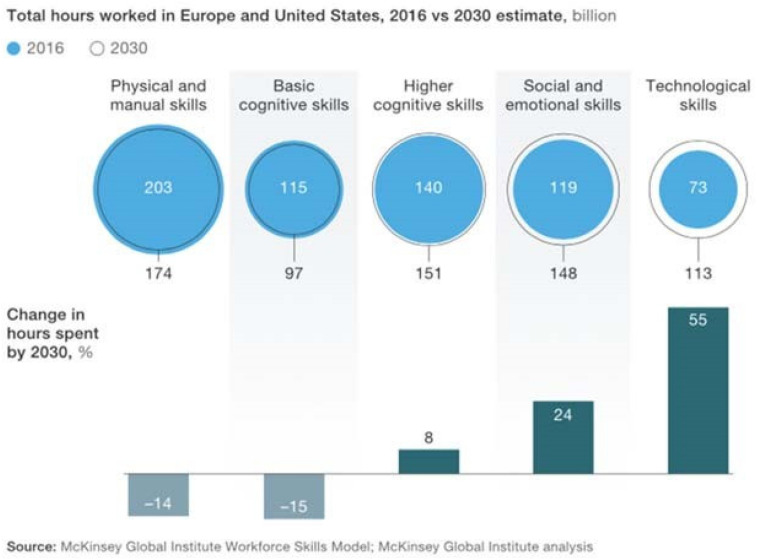
Skill shift caused by automation and AI (Bughin et al., 2018).

The previously mentioned McKinsey study concludes that the need for emotional and social skills as well as technical skills will significantly increase until 2030, while demand for physical and manual skills will decrease (Bughin et al., 2018). They also found advanced technology and programming skills to be the most important skills needed within the next 3 years according to the survey with chief executives. Additionally – as shown in Figure 1. a general shift from activities that require more basic cognitive skills to activities that require higher cognitive skills such as creativity, critical thinking, decision making, and complex information processing can be expected (Bughin et al., 2018).

Furthermore, all tasks that involve interacting intelligently, socially and emotionally with a human counterpart are predicted to continue to require mostly human involvement. Machines have difficulties successfully persuading, negotiating with and caring for others (Arntz et al., 2016). The people in need of the services in question tend to prefer humans over machines to carry out certain tasks in areas such as care for the elderly, childcare or psychological assistance. Machines might start to assist and complement human work, but humans performing certain tasks are still in high demand and considered to hold great societal value (Pratt, 2015).

While there have been advances in the emerging field of “emotion-tracking AI,” that try to recognize and read inner emotions such as pain by a close analysis of text and facial expressions, these technologies have been shown to have made controversial assumptions and to have incorrectly encoded people’s moods, mental health, and even guilt or innocence (Whittaker et al., 2018). These kind of ethical and legal challenges in the context of emotional and social intelligence will make it less likely that these occupations could be fully automated in the near future even if this becomes technically viable.

## Expected Impact on Society

Automation and digitization will not only bring challenges but also offer opportunities, such as new prosperity and higher productivity (Bughin et al., 2018). Some researchers such as those of the New Economics Foundation, a London-based think-tank, additionally believe in sociological benefits of automation such as a potential reduction of the normal working hours to about 20 per week. They believe this could address a range of related problems such as overwork, unemployment or inequalities. In a similar vein, the sociologist Peter Fleming in his “Mythology of Work” proposes a “post-labor” strategy, including a 3-day work-week (Fleming, 2015).

Despite their potential upsides, however, it can already be seen that automation and digitization impact polarization in society (Dengler and Matthes, 2018). Even if large job-losses remain unlikely and employment overall should increase due to innovation, large shifts in required skills, occupations and industries are certain. Automation and digitization will largely impact low qualified workers since they perform a greater share of automatable tasks (Arntz et al., 2016) often involving physical or manual skills. If machines increasingly complement human work, the tasks left for humans to perform will be more complex and demanding. Workers with low education and qualifications might face shrinking employment possibilities (Arntz et al., 2016) while demand for highly skilled workers will increase, leading to an intensified competition for top talent and a growing income gap.

In conclusion, the available research overwhelmingly shows that the more manual and less skilled a type of work is, the higher the probability that it might be made redundant by automation. The biggest societal challenge identified by researchers across studies for the future of work accordingly is to deal with rising inequalities and to provide sufficient re-training and protection to ensure the well-being of the less qualified workforce (Arntz et al., 2016). People are dehumanized when they feel “socially useless” (Kile, 2013) which is why finding solutions to people’s fears of automation and their potential unemployment is so significant. If governments and companies fail to provide solutions to these challenges, the threat of social tensions, political upheavals and violent reactions to unemployment created by mass idleness or inhumane working conditions increases significantly (Kile, 2013).

## Perception of the Workforce

Very little research has been conducted on how employees view their own jobs and careers in the age of these potential changes (Brougham and Haar, 2018). One of the few studies on the perception of the workforce was conducted by the Pew Research Center among United States workers in 2015. Sixty-five percent of the questioned United States workers stated to believe that in the future a large proportion of human labor will be replaced by robots and computers, while at the same time 80 percent expected their own jobs to continue to exist and change little over the next 50 years (Smith, 2016). The Pew study shows clearly that the surveyed workers were aware of the potential of automation but did not feel strongly threatened by its expected changes.

Another study conducted by the German Ministry of Labor and Social Affairs analyzed the impact the workforce was already experiencing by technologies such as AI and how this influenced the workers’ outlook for the future across a range of occupations and industries. Only 13 percent of the surveyed employees in Germany considered it likely that their work would be replaced by a machine in the future (BMAS, 2016) which is fairly in line with the 12 percent risk of automation predicted by Arntz, Gregory and Ziehran for Germany at large. However, in spite of low-skilled workers being objectively more likely to see their work changed or taken over by a machine than higher skilled ones, low-skilled workers did not appear to be proportionally more concerned about the negative effects of automation (BMAS, 2016).

It should also be mentioned that the German governmental study showed that the surveyed workers described experiencing positive effects and opportunities with automation and digitization. Almost 30 percent of employees reported physical relief due to technological innovations, and about a third of employees described experiencing greater freedom of decision-making. More than 50 percent felt that their productivity had increased and almost 80 percent of employees recognized the need to constantly develop their own skills as a result of technological changes (BMAS, 2016).

In conclusion, none of the few studies that examine how the workers currently perceives digitization and automation and how it is preparing for changes, compare their findings to the actual impact of automation and digitization predicted by research and experts. This article consequently aims to close this research gap by determining if and how accurately the workforce is currently able to assess technology’s potential to replace their jobs in comparison with expert predictions and identifying the factors which influence this assessment.

## Hypotheses

In order to assess the perception of the workforce, a set of hypotheses (Table 1) was developed based on the literature review discussed above which showed that the risk of labor being replaced by machines varies greatly according to factors such as workers’ education or type of work. Consequently, the hypotheses state expectations on how demographics and work characteristics could influence workers’ level of fear, perceived opportunity and preparation for a future with automation (H_1_–H_4_). The variables fear of automation and perceived opportunity were selected as indicators for the level of risk workers perceive for their own work in order to then compare these indicators to the risk actually predicted by research and literature.

**TABLE 1:** Hypotheses overview.

**Hypotheses**
H1: The higher the level of *Education*, the lower the *Fear of automation* (H_1a_) and the higher the *Perceived Opportunity* (H_1b_) and *Preparation for the future* (H_1c_)
H2: The higher the *Age*, the lower the *Fear of automation* (H^2a^), *Perceived Opportunity* (H_2b_), and *Preparation for the future* (H_2c_)
H3: The more complex the current work position, the lower the *Fear of automation* (H_3a_) and the higher the *Perceived Opportunity* (H_3b_)
H4: The more manual/physical the tasks of a certain occupation, the higher the *Fear of automation* (H_4a_) and the lower the *Perceived Opportunity* (H_4b_) and *Preparation for the future* (H_3c_)
H5: The higher the *Fear of automation*, the lower the *Perceived Opportunity* (H_5a_) and the higher the extent of the *Preparation for the future* (H_5b_)
H6: The higher the *Perceived Opportunity*, the higher the *Preparation for the future*

Since very little research is available on how fear and perceived opportunity could ultimately affect the degree to which workers prepare, more general concepts were applied for these hypotheses. It is expected that workers showing fear of their work being automated or perceiving opportunities are aware of future trends to a certain extent and therefore more likely to prepare for them than those unaware of potential threats and challenges posed by digital technologies (H_5_ and H_6_) which is why these variables were identified.

## Profile of the Spanish Workforce

Spain has a population of approximately 46.5 million and an economy that is the fifth largest in the European Union and the thirteenth in the world in terms of gross domestic product (GDP). According to the European Commission Spain’s business structure is highly fragmented, consisting of small business units. Most small enterprises operate in the services sector while in contrast, the majority of large companies is concentrated in the industrial sector. Additionally, a great number of large Spanish companies operate in sectors related to infrastructure development, renewable energy, tourism, banking, insurance, the textile industry, health technology, aeronautics, the agri-food sector and the car industry (European Commission, 2020).

The Spanish labor market, however, also involves a set of challenging conditions for its workforce, such as high unemployment rates among young people and works over 50 (European Commission, 2020). Since the peaks in youth unemployment of above 50% in 2012, the youth unemployment rate has been declining (Verd et al., 2019) although in the first quarter of 2020 it continues to be above 30%. The overall unemployment rate is recorded to be 14% (Spanish Labour Force Survey, 2020). Additionally, over-qualification, long-term unemployment, low-skilled persons and a large number of temporary workers pose structural problems for Spain’s labor market (Peiró et al., 2012; European Commission, 2020).

## Sample and Measures

The data was collected through the survey “*Los trabajadores españoles ante la automatización*” – referred to in this article as “the Survey” – which was conducted by the Future for Work Institute^1^ in collaboration with the Universitat Oberta de Catalunya. The Survey which can be found in the Annex questioned a large sample of Spanish workers via Randstad^2^ and via the *Unión General de Trabajadores* (UGT)^3^ on their degree of concern regarding the effects of work automation and their response to this challenge. The group of surveyed individuals – referred to in this article as “workers” – included employees of all levels as well as self-employed individuals with own employees.

The national Survey was fully completed by 1559 Spanish workers between April 2018 and January 2019. A total of 35.2% of respondents stated to be living in the Madrid greater area while 28.2% identified as residents in Cataluña. Males composed 57.4% of the sample and the majority of respondents (about 80%) stated to be between 31 and 55 years old. About 60% of workers had obtained at least a bachelor’s degree as their highest level of educational attainment and respondents’ professional backgrounds were heterogeneous including but not limited to business/sales (20.7%), management (12.4%), engineering (44.7%), and scientific or academic fields (9.3%).

**FIGURE 2:**
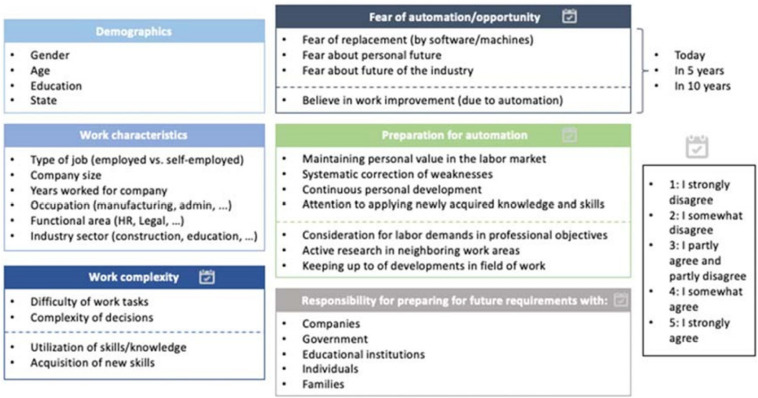
Survey questions by categories.

The Survey consisted of 43 questions in Spanish. The first questions were related to respondents’ demographics as well as their current work position (Figure 2). The respondents were further asked to determine their level of concern over machines and software replacing their jobs and endangering their personal work situation as well as the future of their sector. Another part of the Survey requested that the workers describe their expectations as to how automation might improve their jobs. The answer modality was a five-point Likert scale ranging from “I strongly agree” to “I strongly disagree.” The respondents were finally asked to rate their own measures in preparing for a future with automation for various categories.

## Model Specification

Social science researchers generally use multiple indicators to measure an underlying dimension, i.e., an assumed quantitative construct. Responses to the observable variables (Survey items) are combined to properly compose a global factor or index. When the nature of the observable indicators is continuous, there are two basic approaches for computing these indexes. One can either use the *summated rating scales* (SRS) method by taking the average of the items corresponding to each construct (i.e., attaching equal weights to each item) or attach the appropriate weights that a factor analysis model provides as composites (i.e., attach items’ weights according to the consistency among the items).

Both approaches increase measurement reliability by averaging out random errors, improve precision and discrimination as the factor or composite index global range gets larger and achieves parsimony when making comparisons between different groups. When the nature of the observable indicator is categorical - as is the case here for the work characteristics - analogous approaches are available based also on the consistency (degree of association) among the indicators (Greenacre, 1984; Gifi, 1990; Krzanowski and Marriott, 1994; Batista-Foguet et al., 2004).

A series of *confirmatory factor analysis* (CFA) models were therefore specified in order to summarize the answers to multiple individual questions into single factors representing *Fear of automation*, *Perceived Opportunity*, *Preparation for automation*, and *Work complexity.* Indeed, these CFAs were carried out to test the unidimensionality of each set of items attached to each factor.

Additionally, there is a special interest in the demographic and work-related determinants of these four mentioned dependent factors in the context of our study. In order to summarize the information related to these work-related determinants (work characteristics) into one underlying “*WorkCharact*” factor the following items were considered as *active* variables in a *multiple correspondence analysis* (MCA): *Type of work, Occupation, Functional area*, and *Industry sector*.

These same four variables were used as active in a subsequent cluster analysis to explore potential profiles among the 1559 employees surveyed. We compared different cluster solutions (three to seven) and cluster-profiles^4^ and chose the four-clusters solution based on its plausibility regarding its relationship with (1) the derived *WorkCharact* factor, (2) the supplementary-illustrative demographic variables *Age* and *Education*; and (3) the other three factors *Fear*, *Opportunity*, and *Preparation for automation*. As a result of this clustering process a sequence of profiles according to the employees’ work characteristics was established.

Overall, it was found that those employees whose educational level is the lowest among the entire respondent workforce also show the lowest scores in the continuum of this work characteristics factor. Their profiles correspond to slightly younger males than the average worker and they tend to work in manual labor with elementary occupations and in services functions. These employees are also the ones who perceive the future with the highest degree of *Fear* but only show slightly above average interest in preparing for the future. At the other end of the spectrum lie those employees who stand out for their generally high level of education, showing an overrepresentation of members of the “business owners-entrepreneurs” work type, along with “technical professionals,” “directors and managers” as well as employees working in “HR.” The highest scores for this *WorkCharact* factor are also associated with the lowest level of *Fear* about an automated future and an average interest in preparing for the future.

**FIGURE 3:**
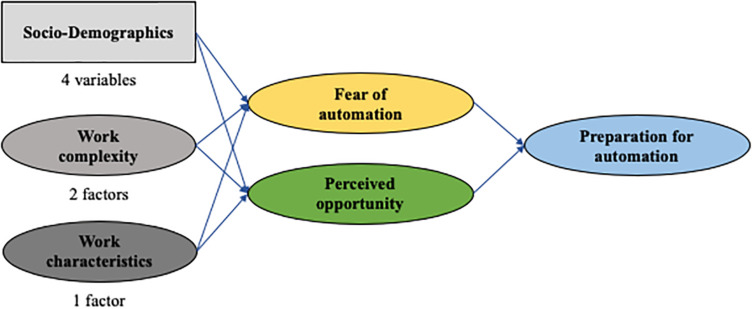
Hypothesized Relationships between Factors.

The path diagram in Figure 3 summarizes our model with the hypothesized relationships among the factors which influence the perception of the workforce with regard to its *Fear of automation, Perceived Opportunity*, and *Preparation* for a digitized future.

## Data Analysis Strategy

First, an *exploratory data analysis* (EDA) was carried out in order to validate whether the underlying assumptions of the *structural equation model* (SEM) in Figure 3 were correct. The first step thereto was to explore and clean the Survey’s data using the statistical computing software R^5^. Only respondents who had answered all questions were considered in order to avoid missing values (*N* = 1559). Supplementary Appendix S6 shows the resulting descriptive statistics for continuous variables and Supplementary Appendix S7 contains exemplary frequency tables for categorical variables.

In the next step, the structural model in Figure 3 was estimated using the maximum likelihood criterium. Following the two-stage approach proposed by Anderson and Gerbing (1988) the measurement model (epistemic relationships) was first tested using a CFA followed by an estimation of the structural model itself using LISREL 9.1. As a result of the first step the convergent and discriminant validity as well as the reliability of measures could be assessed. The second step allowed the testing of the structural relationship between the model’s latent variables (Jöreskog and Sörbom, 2015) thus providing an empirical test for the hypothesized direct and moderating effects.

## Measurement Model

Table 2 shows the goodness-of-fit for the CFA, which tests the measurement model underlying the determined factors. The results confirm the suitability because the covariances among latent variables are not restricted, i.e., they are free to covariate. Table 3 also indicates that all global indexes such as the χ^2^/df ratio, RMSEA, CFI, and SRMR^6^ are within acceptable thresholds (Hu and Bentler, 1999; Hooper et al., 2008; Wessels et al., 2019) and that the structural model coefficients can therefore be estimated.

**TABLE 2:** Global fit indices for the measurement and structural model (*n* = 1559).

**Model**	**SB*χ*^2^(df)**	**RMSEA**	**CIRMSEA**	**PCl**	**SRMSR**	**Missp**	**Missp > 0.10**
Measurement	115 (36)	0.0336	0.0258; 0.0415	1.00	0.0204	1	0
Structural	136 (42)	0.0378	0.0308; 0.0450	0.998	0.0277	1	0
	(a)	(b)	(c)	(d)	(e)	(f)	(g)

*(a) Satorra-Bentler chi square and df; (b) RMSEA; (c,d) confidence interval & probability of close fit for RMSEA; (e) Standardized Root Mean Square Residual; (f) detected misspecifications for a power ≥ 0.8; (g) detected misspecifications greater than Δ = 0.10, for a power ≥ 0.8.*

**TABLE 3:** Bivariate correlations among the seven variables.

	**PrepAut**	**Opportunity**	**Fear**	**Age**	**Educ**	**WorkComp**	**WorkCharact**
PrepAut	1.000						
Opportunity	0.775	1.000					
Fear	−0.039	0.076	1.000				
Age	−0.104	−0.397	−0.013	1.000			
Educ	−0.053	−0.074	−0.093	0.003	1.000		
WorkComp	0.200	0.218	−0.133	0.002	0.103	1.000	
WorkCharact	−0.106	−0.168	−0.145	0.098	0.619	0.118	1.000

## Results

### Structural Model – Direct Effects

**FIGURE 4:**
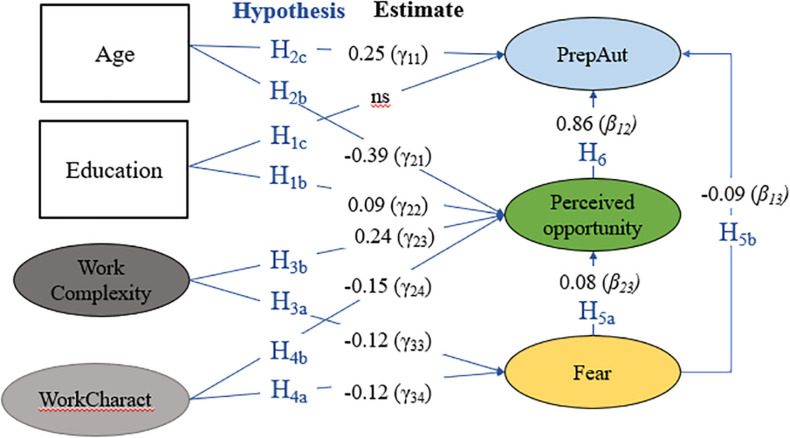
Statistically significant direct effect estimates of structural model.

Once the measurement model had been tested, the Maximum likelihood (ML) method was used on the covariance matrix in order to estimate the direct effects within our structural model, that is, how the respondents’ work factor and demographic characteristics (constructs) affect their *Fear of automation, Perceived Opportunity* and likelihood to *prepare for the future*. The specified model in Figure 4 could not be rejected by any of the global goodness-of-fit statistics despite encountering a situation of high power (attributed to the large sample size and high item reliabilities). The same global test indexes from Table 4 but now corresponding to the structural model (which includes our hypotheses constraining the relationships among the seven factors) actually portrays a better fit than the indexes from the measurement model. Additionally, no additional misspecification errors were identified in the detailed diagnosis.

**TABLE 4:** Statistically significant direct, indirect and total effect estimates for the structural model.

**Dependent Variable**	**Independent Variable**	**Direct effect**	**Indirect effect**	**Total effect**
Preparation	Opportunity	0.859		0.871
	Fear	−0.089	0.056	ns
	Age	0.246	−0.335	−0.091
	Educ	0.087	ns	0.058
	WorkComp	ns	0.217	0.217
	WorkCharact	−0.131	−0.0140	−0.140
Perceived Opportunity	Fear	0.083	–	0.075
	Age	−0.388	ns	−0.409
	Educ	−0.087	−0.09	−0.083
	WorkComp	0.246	−0.009	0.223
	WorkCharact	−0.173	−0.010	−0.125
Fear	Age	ns	–	ns
	Educ	−0.134	–	−0.140
	CompSkill	−0.116	–	−0.126
	WorkCharact	−0.127	–	−0.140

*Notice that the actual magnitude of the *Educ* and *WorkCharact* path coefficients must be estimated separately because of multicollinearity.*

In the structural model that attempts to explain ***Preparation for automation*** as the dependent variable, *Perceived Opportunity* is the most relevant factor with a standardized direct effect of 0.859 and a total effect of 0.871. Therefore, H_6_ cannot be rejected. The direct effect of *Fear* on *Preparation for automation* is rather small but significant and surprisingly negative (−0.089), however, its total effect is non-significant due to the (again surprisingly) positive indirect effect of *Perceived Opportunity* which is why H_5b_ has to be rejected. These unexpected results regarding the negative effect of *Fear* on *Preparation* and its positive effect on *Perceived Opportunity* which contradict our hypotheses (H_5_) will be discussed in detail in the next section. *Age*, while having a positive direct effect (0.243), also has negative indirect effects on *Preparation* which leads to a significant, but rather small negative total effect (−0.091) and no rejection of H_2c_.

Since the *WorkCharact* factor strongly correlates with *Education* (see Table 5) multicollinearity consequences (such as inflated standard errors, unreliable point estimates of effects and surprising significance tests) emerge when both factors are included as exogenous variables in the equation that predicts *Preparation*. In order to estimate the individual effect of each factor, the other one therefore has to be excluded from the structural equation. Following this approach, both factors show statistically significant effects on *Preparation for automation* (*Education* has a positive effect of 0.089 while the effect of *WorkCharact* is negative, −0.131). These results agree with both hypotheses H_1c_ and H_4c._ The factor *Work Complexity* has a non-significant direct effect on *Preparation for automation*, however, shows a total effect of 0.217 which can mostly be attributed to its indirect effect through *Perceived Opportunity* and *Fear*.

**TABLE 5:** Path coefficient estimates for the structural model in Figure 4.

**Dependent variable**	**Independent variable**	***Beta***	***R*^2^**
Preparation (η*_1_*)	Age	0.246	0.640
	Educ (H_1_)	0.087	
	WorkComp	ns	
	WorkCharact	0.55	
	Fear	−0.089	
	Perceived Opportunity (H_2_)	0.859	
Perceived Opportunity (η*_2_*)	Age	0.401	0.252
	Educ (H_1_)	−0.388	
	WorkComp	0.240	
	WorkCharact	−0.148	
	Fear	0.083	
Fear (η*3*)	Age	ns	0.056
	Educ (H_1_)	−0.134	
	WorkComp	−0.116	
	WorkCharact	−0.127	

*Notice that the actual magnitude of the *Educ* and *WorkCharact* path coefficients must be estimated separately because of multicollinearity.*

Looking at ***Perceived Opportunity*** as the dependent variable, one can see that the direct effect of *Fear* is rather small but significant and surprisingly positive (0.083) which is why H_5a_ is rejected. *Age* shows a negative direct effect (−0.388), so H_2b_ cannot be rejected. However, the indirect effects of age on *Perceived Opportunity* are non-significant. For the *WorkCharact* and *Education* variables, the same issue of multicollinearity which involves having to estimate their effects separately persists (see above). Both *WorkCharact* and *Education* show negative but significant direct effects on *Perceived Opportunity* (*Education* −0.087; *WorkCharact* −0.173) when tested separately. These results lead to a rejection of H_1b_ while they are in line with H_4b_. *Work Complexity* further proved to have a significant positive direct effect on the *Perceived Opportunity* (0.246) which agrees with hypothesis H_3b_.

The effect of all four exogenous factors except for *Age* on ***Fear of automation*** is statistically significant. H_2a_ is therefore rejected. In contrast, the effect of the *Work Complexity* factor which has a relatively small negative standardized direct effect of −0.116 on *Fear* is in line with H_3a_. *Education* and *WorkCharact* variables also show statistically significant effects, although as mentioned, they can only be observed if estimated separately due to multicollinearity. Both factors have negative effects of relatively low magnitude: The standardized coefficients are −0.134 for *Education* and −0.127 for *WorkCharact* and do not allow us to reject either H_1a_ or H_4a_.

### Discussion of Results

In the following the results set out above will be discussed in the larger context of the research study and related to the initial hypotheses H1 to H6. Looking at the **first hypothesis** which reflects the impact of education on workers’ perception of automation, results indeed confirm that the higher the level of education, the less afraid workers are of automation which is very much in line with what research predicts. Workers are also more likely to prepare for a future with automation with an increased level of education. However, the higher level of education surprisingly does not entail a correspondingly high level of *Perceived Opportunity* from automation. One possible explanation could be that more educated workers live in contexts that offer them greater work security and training opportunities. Therefore, these workers might already be used to permanently learning and adapting to change and might not see automation as a significant opportunity for improvement in that respect. For them, factors such as professional relationships and social networking might imply greater opportunities.

Regarding the **second hypothesis**, age does not seem to have great influence on how afraid workers are, however, it does significantly influence both their *Preparation for the future* and their *Perceived Opportunity*. It is not surprising that older workers are not preparing as much and are less excited about a future with automation than younger generations since the former are less likely to experience these changes during their remaining working life. Many older workers may – realistically – trust that they will have stopped working before automation strongly affects their profession. Regarding the **third hypothesis**, it is noticeable that work complexity does not seem to impact how likely it is that workers will prepare for the future, however, it more importantly and significantly does seem to affect their *Fear of the future* as well as the opportunities they perceive. The higher the work complexity and the more and diverse skills used, the less afraid workers are and the more frequently they have a positive outlook on a future with automation. This result conforms to what could be expected according to research.

Not surprisingly, the *WorkCharact* factor showed significant effects on all three dimensions of *Fear, Opportunity* and *Preparation* for the **fourth hypothesis**. Various *Workforce* clusters were identified using the *WorkCharact* factor. Those clusters featuring a more manually oriented type of work showed a higher degree of *Fear*. However, respondents in cluster 1 who are “most at risk” according to research, surprisingly also showed a slightly higher inclination to prepare for the future. This was not expected since these workers will usually have less opportunities to acquire and apply new skills. Results for Workforce clusters 3 and 4 also showed that the higher and more technical or complex the occupation, the lower the *Fear* of automation. However, these groups are surprisingly less inclined to prepare for the future, potentially due to the fact that they hold secure job positions that do not necessarily require constant search for new development opportunities or that these opportunities will be provided by their employers anyway. Another explanation might be that the workers in highly specialized positions have less *Fear* because they – quite realistically according to research - do not expect to be replaced and therefore see no necessity to prepare. The detailed Workforce clusters and their significance regarding the effect of work position, industry and sector on the perception of the workforce can be found in Supplementary Appendix S5.

The result of the analysis applicable to the **fifth hypothesis** is that *Fear of automation* surprisingly does not impact the degree of *Preparation* workers undertake. However, it does positively influence a worker’s *Perceived Opportunity* which was quite unexpected in this context. A possible explanation therefore could be that workers who are afraid of losing their positions might start to actively explore alternatives and learn of new opportunities in the context of automation which could create this positive state of mind. The **sixth hypothesis** was also confirmed: the level of workers’ perceiving opportunity very significantly influences the probability of their being motivated to prepare for a future with automation. Workers seem to be more motivated to search for and acquire new skills if they perceive automation as an opportunity rather than a threat. Psychological factors such as a worker’s past experiences as well as employer characteristics such as development opportunities might further influence whether automation is perceived as a threat or an opportunity. Therefore, these same psychological factors and employer characteristics are also likely to impact the degree of preparation workers will undertake.

## Implications

Practical implications for all involved stakeholders may be suggested as a consequence of the results found above. For the working population in general, a clear implication is the need to be more proactive in their own development and the acquisition of new skills.

For labor unions, automation should generally be a key part of any important negotiation they undertake with companies. They should further intensify their efforts in assessing the impacts of technological changes on employment and the necessary investments to retrain employees. The unions need to push for these findings to be incorporated into their collective bargaining agreements with employers. These agreements should also ensure increased training opportunities as well as programs creating digital awareness among workers. In addition, unions could work as active partners in training efforts for those who have recently been laid off - as they already are doing in several European countries (Bughin et al., 2018). The involvement of unions in these cases could be especially important since dismissed workers will no longer have access to internal development opportunities offered by companies.

Employers need to realize that the cost of retraining employees will in the long-term in many cases be lower than the cost of releasing them and having to hire and train new employees. Retraining employees might also be the only possible strategy for many companies, given the fact that the available supply of talent in the market might further decrease in the future. Given this expectation, the provision of sufficient internal retraining measures may be considered the most important part employers can play in the mitigation of the consequences of automation. Companies should provide continuous learning opportunities and instill a culture of lifelong learning and openness to change throughout their organizations – independently of employees’ age or the years they have worked for the company.

The last important stakeholder for which practical implications can be derived from the above results are governments and public institutions. Their aim should be to introduce policies that regulate and guide all other stakeholders in their decisions and actions concerning automation. This could include the creation of tax benefits and other incentives to encourage companies and other stakeholders to invest in human capital. Traditional public as well as private educational institutions should further evolve their contents and form of teaching to a changed workplace with new requirements. STEM (science, technology, engineering, mathematics) as well as social, emotional and creative intelligence skills were found to be underrepresented, especially in Spain (OECD, 2017). Policy makers working with traditional as well as untraditional education providers should consequently address this shortage. Moreover, more importance should be given to critical and systems thinking as well as adaptive learning in overall education systems in order to successfully face the challenges posed by the continuously changing nature of work.

Concluding, this article’s findings show that creating awareness among workers about challenges, but also – and even more importantly - the opportunities offered by automation and continuous training and education on the new requirements of their workplaces, are key in helping workers prepare for the future. Therefore, sensibilization campaigns, and increased educational opportunities regarding digitization and automation initiated by unions, public institutions as well as private entities targeting all ages, but especially older workers could prove essential for society’s readiness for a digitized future.

The current crisis caused by the Covid-19 pandemic is additionally accelerating many automation processes, digital communication and online work. Human labor is being automated at an increased rate in order to decrease infection risk while continuing business operations. Response robots are being deployed for various tasks such as disinfection, delivering medications and food, measuring vital signs, and assisting border controls (Yang et al., 2020). Simultaneously, the pandemic is forcing many people to undertake an immediate effort in order to learn how to work using digital applications from their home. The Covid-19 pandemic has the role of a catalyst that accelerates and augments the effects of a digital economy (Xiarewana and Civelek, 2020) increasing the relevance of the analysis and findings discussed in of this article. The pandemic and its already visible consequences should demonstrate the urgency in addressing challenges and supporting the workforce in their preparation for a digital work future to stakeholders.

## Limitations and Further Research

Firstly, it must be recognized that the word “statistically significant” has been used profusely and leniently throughout the paper. The herein applied data collection method does not meet most of the requirements on which significance and confidence can be based. Notice accordingly that - since our sample was not gathered randomly - the statistical significance of the conclusions of this paper should be understood as having descriptive rather than inferential meaning.

Secondly, the situation of high power in our analysis potentially compromises the *validity* of our *statistical conclusions* (SCV, Following Shadish et al., 2002 terminology). While the magnitude of the sample size of the study increases the degree of statistical power of the results of this paper, the unreliability of some of the chosen measures has the opposite effect. Moreover, the reduced range that a 5-point Likert scale provides limits variability and therefore makes it difficult to distinguish and measure the actual attitudes of the respondents (i.e., less statistical power, see Batista-Foguet et al., 2014; Dawson and Thomson, 2018). Both the 5-point range as well as the related power situation represent significant limitations to the SCV.

Thirdly, we would like to point out a few reservations regarding the construct validity (CV). The herein identified constructs and research is based exclusively on the responses of a part of the Spanish workforce in one survey. One obvious concern therefore relates to the “*Inadequate preoperational explication of constructs*.” By just reviewing the items attached to our four main latent variables - *Fear*, *Perceived Opportunity*, *Preparation for automation*, and *Work Complexity* – it could be questioned whether these items best represent the constructs we attempt to relate, or whether subdimensions could also have an impact or even play more significant roles.

We further need to refer to the use of the term *effects* of factors on one another that are shown in Figures 3 and 4 throughout the text. However, due to this study not having an experimental or quasi-experimental design, the word “*effect*” employed herein is not able to ensure an actual causal effect. Moreover, since the responses were gathered on a voluntary basis and self-reported, this study is also subject to one of the most pervasive challenges to the validity to CV and SCV, being the “*common method bias*” (Batista-Foguet et al., 2014) which likely would imply an upward bias in correlations (Spector, 2006).

Finally, the external validity of our inferences could also be questioned because certain characteristics of the respondent group - and therefore the data sample - are not representative of Spain’s workforce in general. This refers particularly to the level of employment and comparatively high levels of education of the respondents’ group. Further, a variable tracking as to whether respondents were reached via UGT or Randstad was unfortunately not available in the data. This variable should, however, have been included in the Survey since both institutions have access to workers from very different working environments and distinguishing this factor could have allowed the determination of more granular groups of worker and employment types and therefore have proven valuable for the article’s conclusions.

Further research based on the survey data would need to include consideration of possible moderating effects of demographic or current work variables in the relationship between *Fear* and *Preparation for automation.* The possibility cannot be disregarded that only non-significant results were achieved in this relationship due to the inability to conduct in-depth analyses of all potential moderating influences within the scope of this article.

Additionally, we suggest widening the scope in future research and surveying other stakeholders such as (HR) managers and leaders on their perception of automation and the requirements for a prepared workforce. Comparing the assessment of further stakeholders to the results obtained through this study could provide additional understanding and guidance on how to best address workers and prepare society for a changing future of work.

## Conclusion

Various significant dependencies between workers’ characteristics and their work environment affecting their attitude toward automation could be identified in the analyses for this article. The results surprisingly showed that respondents’ level of fear appears to have little influence on their level of preparation for the future. In contrast to this, the results indicate that workers’ perceived opportunity does significantly positively impact their preparation for automation. Furthermore, the characteristics that showed the greatest influence not only on fear but also on preparatory steps taken by respondents are their level of education, work complexity as well as work position (occupation, industry, and sector). Overall, the results of this article could provide a way for researchers and stakeholders to differentiate between types of workers and identify their specific needs more reliably. In order to consequently adequately address these needs and the labor requirements of the future, awareness and opportunities for personal development have to be ensured in the workplace and wherever future workers are educated by all relevant parts of society. We have just begun to witness the onset of the Covid-19 pandemic before finishing this article. Our expectation is that the pandemic will strongly raise awareness of the necessity as well as the advantages and opportunities provided by digital communication and might lead to an increased effort from all of the mentioned stakeholders to introduce digital progress on a wider and faster scale.

## Data Availability Statement

The data analyzed was provided by the Future for Work Institute and their report “Los trabajadores españoles ante la automatización” can be found at https://www.futureforwork.com/assets/uploads/2019/02/Los-trabajadores-españoles-ante-la-automatización.pdf.

## Author Contributions

All authors listed have made a substantial, direct and intellectual contribution to the work, and approved it for publication.

## Conflict of Interest

The authors declare that the research was conducted in the absence of any commercial or financial relationships that could be construed as a potential conflict of interest.

## Footnotes

1 The Future for Work Institute in Barcelona advises companies on the expected changes in work environments (http://www.futureforwork.com).

2 Randstad is one of the largest providers of temporary work and other human resources services. The company operates in over 40 countries and each day more than 500,000 temporary workers find work through Randstad intermediation (https://www.randstad.es/nosotros/).

3 UGT is the trade union with the highest membership in Spain. In addition to performing traditional union work, UGT also acts as a job center and aims to integrate people with difficulty finding employment (http://www.ugt.es/que-es-ugt).

4 We followed a two-step clustering process. First, a hierarchical cluster technique using Ward’s method was used to decide on the number of clusters. Then a more refined four-clusters solution was obtained by using the moving average Cluster (K-means). Please see Supplementary Appendix S5 for a detailed description of the *WorkCharact* continuum obtained with MCA as well as the four profiles derived from the Cluster Analysis.

5 The identified outliers can be attributed to either mistakes while filling out the survey, respondents who (intentionally) did not collaborate or can be considered true outliers. In any case, the outliers were – in accordance with standard methodology for statistical models - excluded from the analysis. Since the models that were specified - CFA and SEM - analyze co/variance or correlation matrices, it is vital to ensure the linearity of relationships. Fortunately, only few outliers (<1%) were identified which can be considered random. The outliers were imputed using the ML EM method.

6 Since distributions deviate from normality the Satorra-Bentler Robust Chi-square test was deemed appropriate as a global goodness-of-fit index. Additionally, the global fit indices recommended by Credé and Harms (2015) were considered, i.e., the RMSEA value plus its 90% CI, the SRMSR, and the CFI. In the next step the recommendations by Saris et al. (2009) more detailed diagnosis indicators were considered in order to avoid a “global model fit tunnel vision” (Kline, 2005) (1) reasonable estimated values in the expected direction, (2) addition of justified correlated specificities, and (3) the assessment of modification indexes and their expected parameter changes, which led to plausible estimates. When the size of any misspecification is negligible (i.e., its “standardized expected parameter change” falls below 0.15) and leads to a great Chi-square increment, it reveals a situation of excessive statistical power (Saris,Satorra, rbom, 1987). The analysis in this study includes such a situation of high power due to a relatively big sample sizerelative to the parsimonious mode and a high reliability of indicators. This situation of high power is illustrated in the standardized expected change between two correlated specificities: TD (1,5) of 0.07 leads to a Δχ^2^ = 26.
