# Supplementary material for: Debating the Future of Work: The Perception and Reaction of the Spanish Workforce to Digitization and Automation Technologies
Source: Front Psychol. 2020 Aug 10;11:1965. doi: 10.3389/fpsyg.2020.01965 (PMC7430553; doi:10.3389/fpsyg.2020.01965)
Supplement: Supplementary file 1 [file Data_Sheet_1.docx]

**Appendices**

# Appendix 1: Share of Workers with High Automatability by OECD Countries

(Arntz, Gregory, and Zierahn 2016)


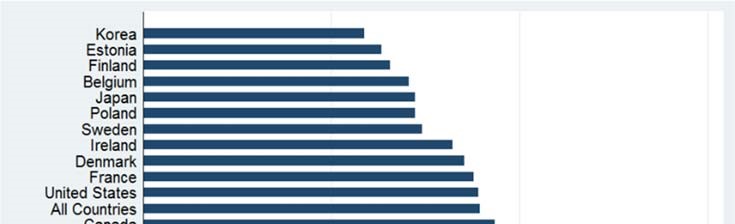

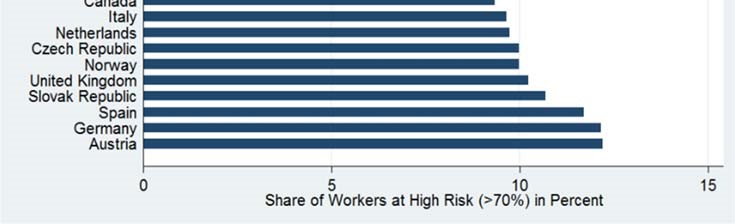


# Appendix 2: Skills mismatch vs. automation and AI adoption (Bughin et al. 2018)


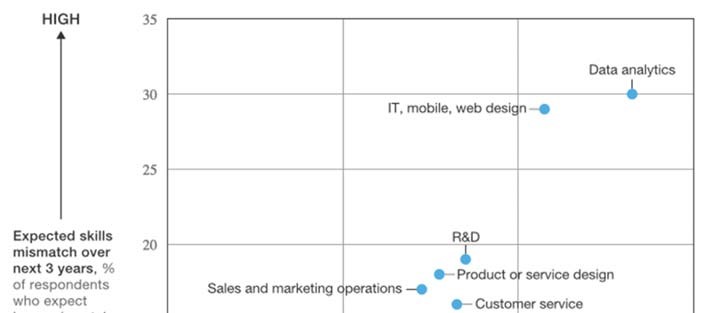

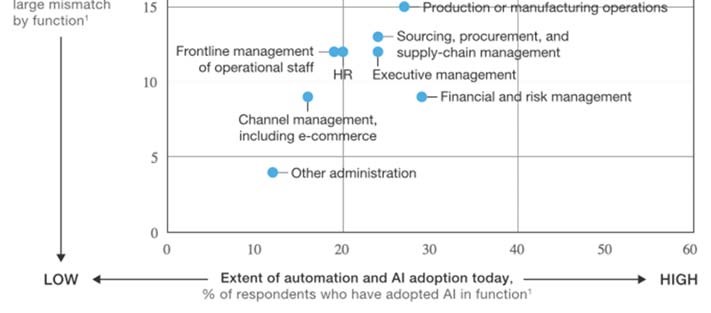


# Appendix 3: US Employment by Risk Category

(Frey et al. 2013)


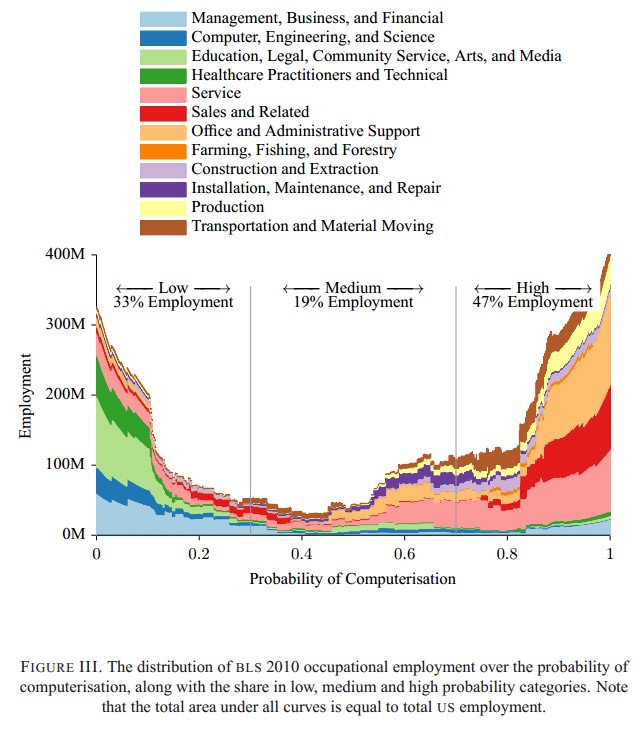


# Appendix 4: Automation potential by major occupation group

(Muro, Maxim, and Whiton 2019)


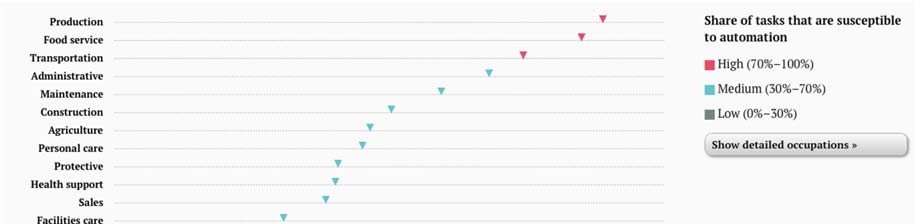

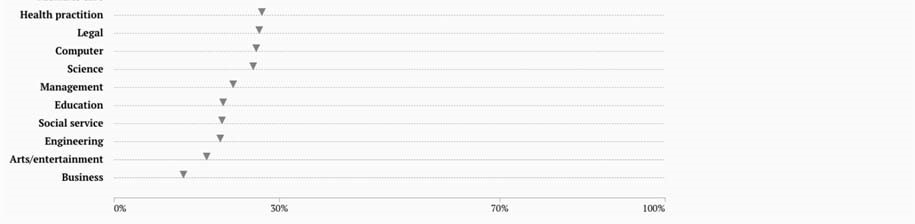


# Appendix 5: Results (WorkCharact factor analysis)

**Used for WorkCharact Factor definition (active variables)**

|  |  |  |  |  | | |
| --- | --- | --- | --- | --- | --- | --- |
| **WorkCharact**  **Factor** | **WorkCharact Label** | **N** | **Type of work** | **Occupation** | **Functional area** | **Industry sector** |
| **1** | **The highest risk and most afraid workers with very low education in production/ manufacturing** | 274 | More employees | Very high risk  (Officers, workers, mechanical tasks and other manual labor, Elementary occupations,  Service and sales) | High risk  (Production/Manufacturin  g, Sales) | Professional scientific and technical activities,  Real estate activities,  Activities of extraterritorial organizations |
| **2** | **The high-risk and afraid workers with low education and professional training in production/ manufacturing** | 375 | Average  (Evenly distributed) | Higher risk  (Military occupations,  Service and sales) | High risk  (Production/Manufacturin  g, Sales) | Fairly even distributed  (Activities of extraterritorial organizations and organizations, Water supply, sanitation activities, waste management and decontamination,  Public administration and defense,  Compulsory Social Security) |
| **3** | **The low-risk scientific and academic professionals with high education who are not afraid but also not preparing for a future with automation.** | 508 | Average  (Evenly distributed) | Low risk  (Scientific and academic professionals) | Lowest risk  (General manager) | Power supply to electricity, gas, steam and air conditioning |
| **4** | **The low-risk, independent workers in human resources with highest education levels who are not afraid and preparing for a future with automation** | 402 | Less employees  (More officer / servant / clerks, freelancers) | Low risk  (Mid-level technicians and professionals, Directors and managers) | Low risk  (Human resources) | Extractive industries,  Sanitary activities and social services |

|  |  |  | |  | **Illustrative variables** | |  | |  |
| --- | --- | --- | --- | --- | --- | --- | --- | --- | --- |
| **WorkCharact**    **Factor** | **WorkCharact Label** | **Preparation** | **Following the news** | **Researching neighboring work areas** | **Considering labor demands for personal objectives** | **Fear** | **Education** | **Gender** | **Age** |
| **1** | **The highest risk and most afraid workers with very low education in production/ manufacturing** | Average /  slightly higher | Average | Average | High | Very high | Very low (high school) | Slightly  more male | Slightly younger (36-45) |
| **2** | **The high-risk and afraid workers with low education and professional training in production/ manufacturing** | Average | Average | Average | Average | High | Low  (professiona l training) | Average | Average (even distribution) |
| **3** | **The low-risk scientific and academic professionals with high education who are not afraid but also not preparing for a future with automation.** | Low | Average | Low | Average | Low | High  (bachelors degree) | Slightly  more male | Very  slightly older (46-50) |
| **4** | **The low-risk, independent workers in human resources with highest education levels who are not afraid and preparing for a future with automation** | Average | High | Average | Average | Very low | Very high (masters degree/doct orate) | Average | Average (even distribution) |

*Table 6: WorkForce clusters overview*

The cluster analysis results showed a possible grouping of the Survey respondents into four WorkForce clusters according to their work attributes. Table 6 provides a summarizing comparison of the below discussed characteristics for all four clusters. The results show that overall, Survey respondents in each WorkForce cluster perceive automation and its realities very differently from the respondents in other clusters.

The **first** Workforce cluster was named “The highest risk and most afraid employees with very low education in production/manufacturing”. This cluster can be characterized by showing very high Fear of automation. Levels of Preparation for cluster 1 are slightly higher than for cluster 2, showing the highest interest in “considering labor demands for personal objectives”. Male respondents as well as workers of the type “employee” are slightly overrepresented, and respondents are generally slightly younger. The level of education is lowest among all clusters with “high school” being mentioned more frequently as the maximum level of education as compared to the average sample population. Workers in cluster 1 predominantly hold occupations involving “officers, workers, mechanical tasks and other manual labor”, “elementary occupations” as well as “service and sales”. The functional areas that are stated most often are “production and manufacturing” as well as “sales”. Frequent industries in this cluster include “professional scientific and technical activities”, “real estate activities” and “activities of extraterritorial organizations”.

The **second** Workforce cluster can be identified as “The high-risk afraid workers with low education and professional training in production/manufacturing”. The typical member of this cluster shows a significant amount of Fear of automation compared to the sample average. Levels of Preparation are similar to the average population for all variables measuring Preparation (“following the news”, “researching neighboring work areas” and “considering labor demands for personal objectives”). cluster 2 further shows even representations for age, gender and type of work. However, the level of education in this cluster is lower than the sample average with workers being overrepresented which state “professional training” to be their maximum education. The occupations most prevalent in cluster 2 are “military occupations” as well as “services and sales” (see Table 6). “Production and manufacturing” as well as “sales” are again the functional areas that are more frequently represented in this cluster, with the industries being fairly evenly distributed.

The **third** Workforce cluster was labeled “The low-risk scientific and academic professionals with high education”. The members of this cluster generally experience little Fear and are not strongly preparing for a future with automation. This cluster can be characterized by its members’ low Fear of automation (even though it is not quite as low as that of respondents in cluster 4) as well as its members’ low interest in preparing for the future, especially in the category of “researching neighboring work areas”. These respondents are further slightly male and older. Members of cluster 3 also show a slightly higher than average level of education with “bachelor’s degree” being strongly represented as the highest level of education obtained. This cluster further shows an even distribution for type of work. The most frequently listed occupations are “scientific and academic professionals” as well as the role “general manager” and the “power supply to electricity, gas, steam and air conditioning” industry.

The **fourth** Workforce cluster can be described as “The low-risk, independent workers in human resources with highest education levels”. Generally, these cluster members stand out for having a comparatively low level of Fear of automation. Its members further show average interest in preparing for the future, only “following the news” slightly more often than respondents in other clusters. These respondents are highly educated, with “master’s degree” and “doctorate” being overly represented as the highest level of education as compared to other clusters. Respondents in cluster 4 show an average distribution for gender and age, but also a higher frequency of independent work types such as “business owners/entrepreneurs” or “freelancers”. The occupations most prevalent in cluster 4 are “mid-level technicians and professionals” as well as “directors and managers”. The functional area that is strongly represented is “human resources” within the “extractive industries” and the “sanitary activities and social services” industries.

The results found for the respondents in *Workforce* cluster 1 and 2 confirm most of our hypotheses and are aligned with current research. This cluster’s high fear of automation score can be ascribed to the specific characteristics of its members. The comparatively low levels of education strongly indicate that these respondents are at considerable risk of being replaced by automation. The main occupations, functional areas and industries represented in cluster 1 and 2 are also largely in line with the occupations and industries most at risk according to literature and research. However, while members of cluster 2 have slightly higher education levels they also show no special interest in preparing for the future. Members of cluster 1 – potentially driven by their higher fear of automation - show the highest overall score for preparation for the future. Stakeholders should therefore target workers with the characteristics of clusters 1 and 2 especially with retraining and preparation measures since these types of workers are most likely to be negatively affected by automation. It is to the advantage of the members of these clusters that they are apparently already quite aware of the necessity of preparation. Due to the currently low level of education as well as high representation of manual work, effective retraining measures are all the more important in order to adapt workers of these clusters to a future in which demand for more complex work will increase.

The results found for the respondents grouped in cluster 3 and 4 generally also confirm the results of current research. As opposed to members of clusters 1 and 2, these respondents stand out for their low fear of automation. They are highly educated and carry out occupations that involve complex and demanding work tasks which according to research makes them less likely to be affected by automation. Members of cluster 4 are not in need of any special attention from stakeholders. These types of workers seem to have had the chance to enter the workforce for employers and in tasks that give them a sense of protection and offer sufficient training and formation opportunities. They show average levels of preparation and tend to follow the news to keep up to date on their work environment. Stakeholders should ensure that members of these clusters have stimulating working environments that offer the opportunity to adapt and develop their skills on an ongoing basis. However, no fundamental change in mindset or attitude is required, since these respondents have the advantage of having the level of education and skill to most likely meet the labor demands of the future.

Members of cluster 3, however, also show little fear of automation but more importantly also the least interest in preparing for automation-related challenges. Respondents are slightly older than those in cluster 4. Cluster 3 should receive some attention from stakeholders since a part of the respondents seem to have a potentially misleading sense of confidence in their work security. This confidence could have detrimental effects for this group of workers because it could keep them from taking adequate preparatory steps. While these workers are less likely to be negatively influenced by automation than members of clusters 1 and 2, they show by far the least incentive to actively prepare. Cluster 3 therefore represents some risk of failure of the workforce to perform the necessary skills adaption since these workers might at first not appear to be at risk and themselves seem unaware of the most serious consequences that automation could have for their futures. If this issue is not adequately addressed, this false sense of security could lead to a gap between the skills demanded and those available in the future labor market.

# Appendix 6: Descriptive statistics (for continuous variables)

| **Descriptive Statistics** | | | | Mean |  |
| --- | --- | --- | --- | --- | --- |
|  | N | Minimum | Maximum |  | Std. Deviation |
| difficulty_work_tasks_11 | 1559 | 1 | 5 | 2.99 | 1.209 |
| complexity_decisions_11 | 1559 | 1 | 5 | 3.02 | 1.215 |
| utilization_skills_knowledge_11 | 1559 | 1 | 5 | 3.38 | 1.315 |
| addition_new_skills_11 | 1559 | 1 | 5 | 3.76 | 1.246 |
| fear_of_automation_today_12 | 1559 | 1 | 5 | 2.08 | 1.250 |
| fear_of_automation_5_years_12 | 1559 | 1 | 5 | 2.58 | 1.336 |
| fear_of_automation_10_years_12 | 1559 | 1 | 5 | 2.96 | 1.459 |
| fear_personal_future_today_13 | 1559 | 1 | 5 | 2.10 | 1.304 |
| fear_personal_future_5_years_13 | 1559 | 1 | 5 | 2.49 | 1.359 |
| fear_personal_future_10_years_13 | 1559 | 1 | 5 | 2.82 | 1.487 |
| fear_industry_future_today_14 | 1559 | 1 | 5 | 2.28 | 1.338 |
| fear_industry_future_5_years_14 | 1559 | 1 | 5 | 2.69 | 1.383 |
| fear_industry_future_10_years_14 | 1559 | 1 | 5 | 3.01 | 1.466 |
| work_improvement_today_15 | 1559 | 1 | 5 | 3.35 | 1.295 |
| work_improvement_5_years_15 | 1559 | 1 | 5 | 3.52 | 1.285 |
| work_improvement_10_years_15 | 1559 | 1 | 5 | 3.63 | 1.313 |
| maintaining_personal_value_16 | 1559 | 1 | 5 | 4.23 | .933 |
| correcting_weaknesses_17 | 1559 | 1 | 5 | 4.24 | .836 |
| personal_development_18 | 1559 | 1 | 5 | 4.32 | .854 |
| applying_new_skills_19 | 1559 | 1 | 5 | 4.38 | .793 |
| considering_labor_demands_20 | 1559 | 1.0 | 5.0 | 3.976 | 1.0118 |
| researching_neighboring_areas_21 | 1559 | 1 | 5 | 3.42 | 1.327 |
| following_news_22 | 1559 | 1 | 5 | 4.06 | 1.020 |
| responsibility_companies_23 | 1559 | 1 | 5 | 4.16 | .932 |
| responsibility_governments_23 | 1559 | 1 | 5 | 3.87 | 1.100 |
| responsibility_educational_institutio ns_23 | 1559 | 1 | 5 | 4.07 | 1.037 |
| responsibility_individual_23 | 1559 | 1 | 5 | 4.38 | .921 |
| responsibility_families_23 | 1559 | 1 | 5 | 3.33 | 1.234 |
| Valid N (listwise) | 1559 |  |  |  |  |

# Appendix 7: Frequency tables (for categorical variables)

|  | | | | **gender_1** | | | | | | |  | | |
| --- | --- | --- | --- | --- | --- | --- | --- | --- | --- | --- | --- | --- | --- |
|  | Frequency | | |  | Percent | | | | Valid Percent | |  | Cumulative Percent | |
| female |  | | | 664 | 42.6 | | | |  | | 42.6 | 42.6 | |
| male |  | | | 895 | 57.4 | | | |  | | 57.4 | 100.0 | |
| Total |  | | | 1559 | 100.0 | | | |  | | 100.0 |  | |
|  | |  | | | | **age_2** | | |  | | |  | |
|  | | Frequency | | | | Percent | | | Valid Percent | | | Cumulative Percent | |
| between 26 and 30 | | 138 | | | | 8.9 | | | 8.9 | | | 8.9 | |
| between 31 and 35 | | 181 | | | | 11.6 | | | 11.6 | | | 20.5 | |
| between 36 and 40 | | 203 | | | | 13.0 | | | 13.0 | | | 33.5 | |
| between 41 and 45 | | 332 | | | | 21.3 | | | 21.3 | | | 54.8 | |
| between 46 and 50 | | 258 | | | | 16.5 | | | 16.5 | | | 71.3 | |
| between 51 and 55 | | 257 | | | | 16.5 | | | 16.5 | | | 87.8 | |
| between 56 and 60 | | 102 | | | | 6.5 | | | 6.5 | | | 94.4 | |
| between 61 and 65 | | 31 | | | | 2.0 | | | 2.0 | | | 96.3 | |
| less than 26 | | 52 | | | | 3.3 | | | 3.3 | | | 99.7 | |
| more than 65 | | 5 | | | | .3 | | | .3 | | | 100.0 | |
| Total | | 1559 | | | | 100.0 | | | 100.0 | | |  | |
|  | | | **education_3** | | | | |  | |  | | |  |
|  | | | Frequency | | | |  | Percent | | Valid Percent | | | Cumulative Percent |
| bachelor degree | | | 563 | | | |  | 36.1 | | 36.1 | | | 36.1 |
| doctorate | | | 41 | | | |  | 2.6 | | 2.6 | | | 38.7 |
| high school | | | 327 | | | |  | 21.0 | | 21.0 | | | 59.7 |
| master degree | | | 316 | | | |  | 20.3 | | 20.3 | | | 80.0 |
| primary education | | | 19 | | | |  | 1.2 | | 1.2 | | | 81.2 |
| professional training or equivalent | | | 291 | | | |  | 18.7 | | 18.7 | | | 99.9 |
| without studies or less than primary | | | 2 | | | |  | .1 | | .1 | | | 100.0 |
| Total | | | 1559 | | | |  | 100.0 | | 100.0 | | |  |

**Annex. Survey - Los trabajadores españoles ante la automatización**

**Introducción (Introduction)**

Esta encuesta ha sido diseñada para investigar la opinión de los trabajadores españoles acerca de la automatización del trabajo.

No le llevará más de 5 minutos. Le agradecemos por anticipado su colaboración.

Los trabajadores españoles ante la automatización

**Segmentación (Segmentation)**

1. **Sexo(*) (Gender)**
   - Hombre
   - Mujer
2. **Edad(*) (Age)**
   - Menos de 26
   - De 26 a 30
   - De 31 a 35
   - De 36 a 40
   - De 41 a 45
   - De 46 a 50
   - De 51 a 55
   - De 56 a 60
   - De 61 a 65
   - Más de 65
3. **Nivel de estudios terminados(*) (Education)**

- Sin estudios o menos que primaria
- Educación primaria
- Enseñanza secundaria / ESO / Bachillerato
- Formación profesional o equivalente
- Grado universitario / licenciaturas / ingenierías
- Maestría / máster
- Doctorado

1. **Comunidad autónoma de residencia(*) (State)**

- Andalucía
- Aragón
- Asturias
- Baleares
- Canarias
- Cantabria
- Castilla La Mancha
- Castilla y León
- Cataluña
- Ceuta
- Comunidad Valenciana
- Extremadura
- Galicia
- La Rioja
- Madrid
- Melilla
- Murcia
- Navarra
- País Vasco

1. **Tipo de trabajador(*) (Type of work)**

- Empleado / trabajador por cuenta ajena
- Autónomo / freelancer
- Propietario de negocio / empresario
- Cooperativista
- Funcionario

1. **Número de empleados de su empresa(*) (Company size)**

- Hasta 10 trabajadores
- De 11 a 49 trabajadores
- De 50 a 249 trabajadores
- De 250 a 1250 trabajadores
- Más de 1250 trabajadores
- N/A (ej. autónomos)

1. **¿Cuántos años lleva trabajando en su empresa?(*) (Years with company)**

- Menos de 1 año
- Entre 1 y 2 años
- Entre 2 y 5 años
- Entre 5 y 10 años
- Entre 10 y 20 años
- Más de 20 años
- N/A

1. **Ocupación(*) (Occupation)**

- Ocupaciones militares
- Directores y gerentes
- Profesionales científicos e intelectuales
- Técnicos y profesionales de nivel medio
- Personal de apoyo administrativo
- Trabajadores de los servicios y vendedores de comercios y mercados
- Agricultores y trabajadores calificados agropecuarios, forestales y pesqueros
- Oficiales, operarios y artesanos de artes mecánicas y de otros oficios
- Operadores de instalaciones y máquinas y ensambladores
- Ocupaciones elementales

1. **Área funcional en la que trabaja(*) (Functional area)**

- Dirección General
- Comercial / Ventas
- Marketing
- Producción / Fabricación
- Operaciones / Compras / Logística / Calidad
- Administración / Contabilidad / Finanzas
- Tecnología y Sistemas de Información
- Servicio a Clientes
- Investigación y Desarrollo / Innovación
- Recursos Humanos
- Legal
- Otros servicios profesionales

1. **Sector de actividad de la empresa(*) (Industry sector)**

- Agricultura, ganadería, silvicultura y pesca
- Industrias extractivas
- Industria manufacturera
- Suministro de energía eléctrica, gas, vapor y aire acondicionado
- Suministro de agua, actividades de saneamiento, gestión de residuos y descontaminación
- Construcción
- Comercio al por mayor y al por menor
- Transporte y almacenamiento
- Hostelería
- Información y comunicaciones
- Actividades financieras y de seguros
- Actividades inmobiliarias
- Actividades profesionales, científicas y técnicas
- Actividades administrativas y servicios auxiliares (alquileres, apoyo al empleo, agencias turísticas, seguridad, servicios a edificios, administración empresas)
- Administración Pública y defensa; Seguridad Social obligatoria
- Educación
- Actividades sanitarias y de servicios sociales (excepto actividades veterinarias)
- Actividades artísticas, recreativas y de entrenimiento
- Otros servicios
- Actividades de los hogares como empleadores de personal doméstico; actividades de los hogares como productores de bienes y servicios para uso propio
- Actividades de organizaciones y organismos extraterritoriales

**Cuestionario (Questionnaire)**

**------ WORK COMPLEXITY (11)**

1. **Pensando en su trabajo actual, responda a las siguientes cuestiones en una escala del 1 (muy poco) al 5 (mucho):(*)**

**(Difficulty of work tasks, complexity of decisions, utilization of new skills, acquisition of new skills)**

¿Recibe tareas que son extraordinarias y particularmente difíciles? (1-5)

¿Tienen que tomar a menudo decisiones muy complicadas en su trabajo? (1-5)

¿Puede utilizar todo su conocimiento y habilidades en su trabajo? (1-5)

¿Puede aprender cosas nuevas en su trabajo? (1-5)

**------ FEAR (12 - 14)**

1. **Me preocupa que lo que hago ahora en mi trabajo puede ser reemplazado por máquinas o software(*)**

# (Fear of replacement)

- Muy en desacuerdo
- Algo en desacuerdo
- En parte de acuerdo, en parte en desacuerdo
- Algo de acuerdo
- Muy de acuerdo

1. **Me preocupa personalmente mi futuro en mi organización debido a que las máquinas o el software reemplacen a los trabajadores(*)**

**(Fear about personal future)**

- Muy en desacuerdo
- Algo en desacuerdo
- En parte de acuerdo, en parte en desacuerdo
- Algo de acuerdo
- Muy de acuerdo

1. **Me preocupa el futuro del sector en el que trabajo debido a que las máquinas o el software reemplacen a los trabajadores(*)**

**(Fear about future of the industry)**

- Muy en desacuerdo
- Algo en desacuerdo
- En parte de acuerdo, en parte en desacuerdo
- Algo de acuerdo
- Muy de acuerdo

**------ OPPORTUNITY (15)**

1. **Creo que la automatización puede mejorar mi trabajo(*)**

**(Believe in work improvement due to automation)**

- Muy en desacuerdo
- Algo en desacuerdo
- En parte de acuerdo, en parte en desacuerdo
- Algo de acuerdo
- Muy de acuerdo

**------ PREPARATION FOR THE FUTURE (16 - 22)**

1. **Me responsabilizo de mantener mi valor en el mercado de trabajo.(*)**

**(Maintaining personal value in the labor market)**

- Muy en desacuerdo
- Algo en desacuerdo
- En parte de acuerdo, en parte en desacuerdo
- Algo de acuerdo
- Muy de acuerdo

1. **Intento ir corrigiendo mis debilidades de un modo sistemático. (*)**

**(Systematic correction of weaknesses)**

- Muy en desacuerdo
- Algo en desacuerdo
- En parte de acuerdo, en parte en desacuerdo
- Algo de acuerdo
- Muy de acuerdo

1. **Me centro en desarrollarme a mí mismo/a de modo continuo.(*)**

**Muy en desacuerdo**

**(Continuous personal development)**

- Muy en desacuerdo
- Algo en desacuerdo
- En parte de acuerdo, en parte en desacuerdo
- Algo de acuerdo
- Muy de acuerdo

1. **Dedico atención de modo consciente a aplicar los conocimientos y las capacidades que he adquirido recientemente.(*)**

**(Attention to applying newly acquired knowledge and skills)**

- Muy en desacuerdo
- Algo en desacuerdo
- En parte de acuerdo, en parte en desacuerdo
- Algo de acuerdo
- Muy de acuerdo

1. **Al formular mis objetivos profesionales, tengo en cuenta la demanda en el ercado de trabajo.(*)**

**(Consideration for labor demands in professional objectives)**

- Muy en desacuerdo
- Algo en desacuerdo
- En parte de acuerdo, en parte en desacuerdo
- Algo de acuerdo
- Muy de acuerdo

1. **Durante el año pasado, me impliqué activamente en investigar áreas laborales cercanas para ver dónde podría tener éxito.(*)**

**(Active research in neighboring work areas)**

- Muy en desacuerdo
- Algo en desacuerdo
- En parte de acuerdo, en parte en desacuerdo
- Algo de acuerdo
- Muy de acuerdo

1. **Durante el año pasado, me mantuve al día de novedades en mi ámbito de trabajo.(*)**

**(Keeping up to of developments in field of work)**

- Muy en desacuerdo
- Algo en desacuerdo
- En parte de acuerdo, en parte en desacuerdo
- Algo de acuerdo
- Muy de acuerdo

1. **¿De quién crees que es la responsabilidad de que las personas consigan adaptarse a los cambios que puede experimentar el trabajo en los próximos años?(*)**

**(Responsibility for preparing for future requirements: Companies, governments, educational institutions, individuals, families)**

- De las empresas- **Companies**

- Muy en desacuerdo
- Algo en desacuerdo
- En parte de acuerdo, en parte en desacuerdo
- Algo de acuerdo
- Muy de acuerdo

- De la administración /gobierno- **governments**

- Muy en desacuerdo
- Algo en desacuerdo
- En parte de acuerdo, en parte en desacuerdo
- Algo de acuerdo
- Muy de acuerdo

- De las instituciones educativas- **educational institutions**

- Muy en desacuerdo
- Algo en desacuerdo
- En parte de acuerdo, en parte en desacuerdo
- Algo de acuerdo
- Muy de acuerdo

- De cada persona- **individuals**

- Muy en desacuerdo
- Algo en desacuerdo
- En parte de acuerdo, en parte en desacuerdo
- Algo de acuerdo
- Muy de acuerdo

- De las familias- **families**

- Muy en desacuerdo
- Algo en desacuerdo
- En parte de acuerdo, en parte en desacuerdo
- Algo de acuerdo
- Muy de acuerdo
